# Patients’ and physicians’ disagreement on patients’ understanding of clinical cancer trial information: a pairwise pilot study of mirroring subjective assessments compared with objective measurements

**DOI:** 10.1186/s13063-019-3416-2

**Published:** 2019-05-29

**Authors:** Pia Dellson, Christina Carlsson, Mef Nilbert, Helena Jernström

**Affiliations:** 1 Department of Clinical Sciences in Lund, Division of Oncology, Lund University and Skåne University Hospital, Barngatan 4, 221 85 Lund, Sweden; 2 Clinical Research Center, Copenhagen University Hospital, Hvidovre, Denmark; 3 Danish Cancer Society Research Center, Copenhagen, Denmark

**Keywords:** Cancer, Clinical study, Comprehension, Informed consent, Medical oncology, Patient understanding, Questionnaire

## Abstract

**Background:**

Informed consent is a prerequisite for patients included in clinical trials. Trial design, inclusion criteria and legal requirements are increasingly complex. This complexity challenges design and delivery of written and oral trial information to ensure understandable information. To evaluate the level of concordance between patients’ and informing physicians’ assessments regarding patient understanding of trial information, we carried out a study based on paired questionnaire data from patients and their physicians. These assessments of patient understanding were further correlated with patients’ factual knowledge of the information provided.

**Methods:**

This pilot study included patients and physicians immediately after the patients had received information on one of 23 ongoing phase III randomised cancer trials at two Swedish sites. In total, 46 patients and 17 physicians contributed data based on two new questionnaires with seven mirroring questions, where concordance was analysed with McNemar’s test. These assessments of patients’ self-estimated understanding were further correlated with the Patient Understanding of Research (Q-PUR) questionnaire that assesses factual knowledge of the information provided.

**Results:**

For each question, 47–61% of the patient–physician pairs were in concordance regarding their assessments of patients’ ‘fully understanding’ or ‘not fully understanding’ various aspects of the trial information. For the discordant pairs, the physicians rated patient understanding lower than the patients themselves, for all seven questions. This difference was significant for five of the questions (*P* ≤ 0.017). The median Q-PUR knowledge score was 11 out of 12, but this score did not significantly correlate with the assessments, either from patients or from physicians.

**Conclusions:**

This study demonstrated a trend for physicians to rate the level of understanding of trial information among potential trial patients lower than the patients themselves. Application of Q-PUR revealed high knowledge scores, but without correlation to the assessments. These findings need validation in an independent setting, with an improved instrument with mirroring questions, and a better-matched measurement of patients’ factual knowledge. These results suggest that physicians need to improve their ability to assess patient understanding of clinical trial information, in order to be able to tailor the patients’ information individually.

## Background

Clinical trials are a mainstay of medical research and crucial to improving future health care services. Prior to inclusion into a clinical trial, the patient must provide informed consent, defined in the Declaration of Helsinki [1]. The purpose of the oral and written patient information is to convey information that enables the patient to reach a level of understanding from which a decision can be taken based on study design, impact and risk-benefit combined with the patient’s individual preferences. In oncology as well as in other medical specialties, however, the trial information is often extensive and its communication is a complex process, which may indeed counteract the actual intent of complete information and full understanding [2–4].

The main responsibility for informing the patient lies with the clinical trial team, which typically consists of the responsible physician and a research nurse. To optimise the possibility for truly informed consent, information needs to be tailored to the individual patient. Proper tailoring of information requires assessment of patients’ understanding of the oral and written information [5, 6]. However, there is limited information on how accurate physicians’ perceptions are with respect to patients’ understanding. A study by Olson and Windish [7] suggested that significant discrepancies exist between how patients and physicians rated patient knowledge, with physicians overestimating patient knowledge. Whether this is also the case with clinical trial information is, to our knowledge, unknown.

A large number of attempts have been made to improve patients’ understanding of both written and oral clinical trial information, as reviewed in [8–11]. The three main forms of interventions tested are multi-media, enhanced consent forms, and prolonged consent discussions. Multi-media interventions have only been shown to improve patient understanding to a limited degree, whereas enhanced consent forms and prolonged consent discussions were deemed to be the most effective interventions [8, 9]. However, in many studies the development process of interventions and outcome measures were poorly described [10]. Thus, more research is warranted on these topics. Several studies have shown that patients overestimate their factual knowledge about clinical trials. In a study by Biedrzycki [12] 72% of the patients answered ‘yes’ to the question ‘Do you basically understand the research study?’ but only 35% had adequate actual research knowledge as measured by the Seven-Item Knowledge Scale, developed by Ellis et al. [13].

Another study, by Joffe et al. [14], used the validated instrument ‘Quality of Informed Consent’ (QuIC), which measures both perceived understanding and factual knowledge about clinical trials, with questions directed to patients. This study found a weak but statistically significant correlation between patients’ factual knowledge and their self-assessment scores, measured by 14 questions. Furthermore, in the study another questionnaire was sent to each patient’s physician, including one question on the physician’s estimate of the patient’s understanding. Here, a weak but statistically significant correlation was found between physicians’ rating of the patient’s understanding and the patient’s factual knowledge. This is, to our knowledge, the only study that carried out pairwise comparisons between patient and physician ratings of patient understanding of clinical trials and their correlation with the patient’s factual knowledge.

To our knowledge, there is a lack of direct comparisons between patients’ own assessments of their understanding of the clinical trial information and physicians’ assessments of their patients’ understanding. The main purpose of this exploratory pilot study was to investigate how patients invited to take part in a clinical cancer trial rated their own understanding of the clinical trial information, and to compare the results with the paired physician assessments of patient understanding. A secondary purpose was to correlate these assessments with patients’ factual knowledge of the oral and written clinical trial information provided.

## Methods

### Setting, participants, and data collection

Between January 2009 and February 2013, 46 paired questionnaires for patients and physicians were collected, and 100% of the distributed questionnaires were returned. There was no recording of patients or physicians declining to participate when invited. Forty-six patients and 17 physicians were included. Each physician could participate a maximum number of five times in the study. The study was conducted at two Swedish sites, Lund and Malmö. Patients who could read and speak Swedish and who were eligible for 23 selected clinical cancer trials were included. The selection criteria for the clinical trials were that the trials should be randomised phase III trials that were expected to include patients for at least one year. Furthermore, the trial had to have a research nurse responsible for handing out the questionnaires. The trials concerned treatment for 14 different cancer diagnoses with both curative and palliative intention (Table 1). It was not recorded which trial the patient was offered, or whether they accepted or declined participation.

**Table 1:** Eligible phase III clinical trials

Study name	Clinical trial number	Diagnosis	Intention	Treatment modality
ACT	NCT00598156	Colorectal cancer	Palliative	Kinase inhibitor
ACT-1	NCT00646854	T-cell lymphoma	Curative	Antibody
BEATRICE	NCT00528567	Breast cancer	Curative	Antibody
BRCATrial	NCT00321633	Breast cancer	Palliative	Chemotherapy
COMPARZ	NCT00720941	Renal cell carcinoma	Palliative	Kinase inhibitor
CYCLUS	NCT00300729	Non-small cell carcinoma	Palliative	Nonsteroidal anti-inflammatory drug
ENESTg1	NCT00785785	Gastrointestinal stromal tumour	Palliative	Kinase inhibitor
ESPAC	NCT00058201	Pancreatic cancer	Curative	Chemotherapy
EXAM	NCT00704730	Thyroid cancer	Palliative	Kinase inhibitor
HYPO-RT-PC	ISRCTN45905321	Prostate cancer	Curative	Radiation therapy
INTORSECT	NCT00474786	Renal cell carcinoma	Palliative	Kinase inhibitor
MAIN	NCT00486759	B-cell lymphoma	Curative	Antibody
OVAR 12	NCT01015118	Ovarian cancer	Palliative	Kinase inhibitor
OVAR 16	NCT01462890	Ovarian cancer	Palliative	Antibody
PALETTE	NCT00753688	Sarcoma	Palliative	Kinase inhibitor
PANTHER	NCT00798070	Breast cancer	Curative	Chemotherapy
PRELUDE	NCT00332202	B-cell lymphoma	Curative	Kinase inhibitor
RASTEN	NCT00717938	Small cell lung cancer	Palliative	Low molecular heparin
SOFT	NCT00066690	Breast cancer	Curative	Endocrine
SOLD	NCT00593697	Breast cancer	Curative	Antibody
SPRINT	NCT00875667	Mantle cell lymphoma	Palliative	Immune modulator
START	NCT00409188	Non-small cell carcinoma	Palliative	Vaccine
SUN1120	NCT00676650	Prostate cancer	Palliative	Kinase inhibitor

Informed consent was obtained from all participants. The study was approved by the Lund University Ethics Committee (Dnr 346/2007).

A study design with paired questionnaires was used, one questionnaire for the patient receiving information and another questionnaire for the informing physician. The patients completed the questionnaire directly after they had received the oral and written patient information on the clinical cancer trial they were about to consider participation in. The physicians completed their questionnaire directly after the consultation when they had informed the patient about the clinical trial.

### Questionnaires

The study comprised two questionnaires, one for patients and one for physicians. The patient questionnaire was composed of the following three sections: 1) demographic information; 2) patients’ assessment of perceived understanding, seven mirroring questions (7Q-PAT); and 3) patients’ factual knowledge measured by the Patient Understanding of Research questionnaire (Q-PUR). The physician questionnaire was composed of the following two sections: 1) demographic information; and 2) physicians’ assessment of their patients’ understanding, seven mirroring questions (7Q-PHYS).

The demographic information included gender and age. Information from additional questions about educational level and previous participation in a clinical trial by the patient or their next of kin was also collected from patients. Physicians were asked about the number of years spent working in oncology, type of post, and number of patients they had included in a clinical trial. Each physician could participate a maximum number of five times in the study, to avoid any single individual dominating the study material.

The section on perceived patient understanding of oral and written information was constructed by the research team and consisted of seven mirroring questions for patients and physicians, respectively (7Q-PAT and 7Q-PHYS; Table 2). The seven questions on perceived patient understanding were selected based on earlier studies where key points for consideration were identified [15–17]. The questions included separate queries on how the patients understood the written and oral trial information respectively. During the process of designing the questionnaire, the seven questions were tested on research nurses and physicians and adjusted according to their feedback. The questions concerned how the patients rated their understanding of the oral and written information, respectively, of the terms and expressions used by the physicians orally and in the written information, of the study treatment and follow-up, of the side effects, and of the aim of the clinical trial (Table 2). Each question was formulated as a statement with four possible answers: fully agree/partly agree/do not fully agree/disagree.

**Table 2:** Patient and physician responses to seven statements regarding the patient’s understanding of the information

		Patient responses					Physician responses				
	Patient/physician questions	Fully agrees n (%)	Partly agrees n (%)	Does not fully agree n (%)	Disagrees n (%)	n	Fully agrees n (%)	Partly agrees n (%)	Does not fully agree n (%)	Disagrees n (%)	n
1.	I/the patient understood the oral information completely	38 (82.6)	8 (17.4)	-	-	46	26 (56.5)	20 (43.5)	-	-	46
2.	I/the patient understood the written information completely	37 (80.4)	9 (19.6)	-	-	46	23 (51.1)	21 (46.7)	1 (2.2)	-	45
3.	I/the patient understood all words and expressions that the physician used during the oral information	36 (78.3)	8 (17.4)	2 (4.3)	-	46	24 (52.2)	22 (47.8)	-	-	46
4.	I/the patient understood all words and expressions in the written information	30 (65.2)	13 (28.3)	3 (6.5)	-	46	19 (42.2)	24 (53.3)	2 (4.4)	-	45
5.	I/the patient understood how the treatment would be carried out and followed up	42 (91.3)	4 (8.7)	-	-	46	30 (65.2)	16 (34.8)	-	-	46
6.	I/the patient understood what side-effects the treatment may have	40 (88.9)	4 (8.9)	1 (2.2)	-	45	28 (60.9)	18 (39.1)	-	-	46
7.	I/the patient understood the purpose of the research study	40 (87.0)	5 (10.9)	1 (2.2)	-	46	33 (71.7)	10 (21.7)	3 (6.5)	-	46

In the patient questionnaire, but not in the physician questionnaire, a third section that measured the patients’ factual knowledge of clinical trial information was included. For this section the validated Patient Understanding of Research questionnaire (Q-PUR) [18] was used. Q-PUR is a multiple-choice instrument that objectively tests the patients’ factual knowledge about clinical trials [18]. The 12 Q-PUR questions are presented in Table 3. For the complete Q-PUR instrument, see the original paper by Hutchison [18]. Regarding reliability, Q-PUR has been shown to have an overall questionnaire score with an acceptable Cronbach’s alpha of 0.77 (95% CI 0.69–0.84). The corrected item total correlation between individual questions and the overall questionnaire score is, however, low (< 0.4) for several of the questions. Six of the 12 questions had an item total correlation above 0.4 and were deemed to most reliably measure patient understanding. These six selected questions are indicated in Table 3. Separate analyses of these six questions were also performed.

**Table 3:** Results for Q-PUR and indication of six and three selected questions

	Q-PUR question	Percentage of correct answers	6 questions	3 questions
1.	The main reason for carrying out research with patients is…	93.5		x
2.	Research with patients is carried out…	91.3	x	
3.	In a *randomized* clinical research trial/study…*	95.7	x	
4.	The main aim of a randomized trial is to…	97.8		x
5.	When a trial is randomized…	87.0	x	
6.	It is justified for doctors to carry out a randomized trial…	65.2		x
7.	If ‘best supportive care’ or ‘symptom control’ is one of the randomization options in the trial, it means that…**	65.2		
8.	Patients are chosen for a trial…	82.6	x	
9.	Taking part in the trial…	95.7	x	
10.	You can leave a trial…	95.7		
11.	If you do not want to take part in a trial…	87.0	x	
12.	Doctors involved in clinical research trials/studies…	65.2		

* In the Swedish translation the following statement was inserted after the word randomized: ‘(the patients are assigned by lot to one group or the other)’

** In the Swedish translation the words ‘best supportive care’ in English were kept in parentheses after the Swedish translation, and the words ‘symptom control’ were omitted

Question number 7 in the 7Q-PAT and 7Q-PHYS read ‘I/the patient understood the purpose of the research study’. The corresponding questions measuring the purpose of the study in Q-PUR were questions number 1, 4 and 6 (Table 3). In order to specifically study how well the questionnaires correlated regarding these questions on study purpose, separate analyses were performed between question number 7 in the 7Q-PAT and 7Q-PHYS and questions number 1, 4 and 6 in Q-PUR. The 12 Q-PUR questions were translated to Swedish and re-translated to English by professional translators to ensure that the questionnaire would be correctly represented in Swedish.

### Statistical analysis

The data were analysed using SPSS version 24.0.0.1 (IBM). For the demographic data, the number and percentages for each group are presented for the categorical variables. The continuous variables, i.e. participants’ age and number of years working as physicians in oncology, are presented with median and range. For the physicians, who may have included up to five patients over the study duration of five years, the demographic data from the first questionnaire are presented.

For the rating of patients’ perceived understanding (7Q-PAT and 7Q-PHYS), each question could be answered as one of four categories. Few participants indicated ‘do not fully agree’ and none indicated ‘disagree’. Therefore, the data were dichotomised according to ‘fully agree’ (yes/no). If either the patient or the physician in a pair had not answered a question, the pair was discarded. McNemar’s test was used to evaluate the concordance for ‘fully agree’ between each patient–physician pair for each of the seven questions in the 7Q-PAT and 7Q-PHYS. In case of missing data for either patient or physician, both were excluded from the pairwise analysis.

For the 12 Q-PUR questions on patients’ factual knowledge, the percentage of patients indicating the correct answer was calculated. Spearman rank correlations between the total number of correct answers for the 12 questions and ‘fully agree’ on the seven questions in the 7Q-PAT and 7Q-PHYS were calculated for patients and physicians, respectively. Furthermore, the six Q-PUR questions with the highest item total correlation according to the original paper [18] and the three Q-PUR questions concerning the purpose of a clinical trial were correlated with the number of ‘fully agree’ for the seven questions in the 7Q-PAT and 7Q-PHYS of perceived understanding for patients and physicians, respectively.

The three Q-PUR questions concerning the purpose of a clinical trial were selected for a separate analysis in relation to question number 7 (‘I/the patient understood the purpose of the research study’) regarding perceived understanding of the purpose of the trial. Spearman rank correlation was used to analyse the correlation between number of correct answers for the three Q-PUR questions and whether or not the patients and their physicians indicated that the patient ‘fully’ understood the purpose of the trial. In addition, McNemar’s test was used to analyse concordance between patients who ‘fully’ understood the purpose of the trial (question number 7 in the 7Q-PAT) and scored three correct answers on the three Q-PUR questions. Similarly, a second McNemar’s test was used to analyse concordance between physicians who indicated that their patients ‘fully’ understood the purpose of the trial (question number 7 in the 7Q-PHYS) and whether or not the paired patients scored three correct answers on the three Q-PUR questions.

The demographic factors of the patients were compared with the median Q-PUR score for the 12, six and three questions (Table 3). The dichotomous variables (sex, previous participation in research trial either self-experienced or via next of kin) were compared with Mann-Whitney U-test. Age was categorised in three groups (< 50, 50–64 and ≥ 65 years). Median Q-PUR scores were compared between age groups and different levels of education with Jonckheere-Terpstra Test for Trend.

No formal a priori power calculation was carried out. A pilot study with a minimum of 40 pairs was considered large enough to reliably quantify correlations and associations between the various questions regarding both patients and physicians. Statistical significance was set at *P* < 0.05. All *P* values were two-tailed. Since this is an exploratory study, each *P* value should be viewed as the level of evidence against each null hypothesis. Therefore, nominal *P* values without adjustment for multiple testing are presented [19].

## Results

The study included 46 patients, who were informed by 17 physicians. See Table 4 for characteristics of patients and physicians in the study.

**Table 4:** Patient and physician characteristics

		Patients (*n* = 46)	Physicians (*n* = 17)
		Number (%) or median (range)	Number (%) or median (range)
Gender	Female	37 (80.4%)	5 (29.4%)
	Male	9 (19.6%)	12 (70.6%)
Age	Median age, years (range)	62 (21–76)	51 (32–65)
Educational level	Compulsory school (1–9 years)	7 (15.2%)	0 (0%)
	Senior high school or vocational training (10–12 years)	16 (34.8%)	0 (0%)
	College or university (13–16 years)	23 (50.0%)	17 (100%)
Participation in clinical trial	Previous participation in a clinical trial	6 (13.0%)	–
	Next of kin has participated in a clinical trial	4 (8.7%)	–
Years working in Oncology	Years (range)	–	17 (3–35)
Working as	Resident physician	–	2 (11.8%)
	Specialist in oncology	–	15 (88.2%)
Number of patients included in clinical trials	< 10	–	4 (23.5%)
	10–100	–	11 (64.7%)
	> 100	–	2 (11.8%)

Table 2 shows the patient and physician responses to the seven statements regarding the patients’ perceived understanding of clinical trial information (7Q-PAT and 7Q-PHYS). On a group level, between 65.2% and 91.3% of the patients fully agreed with each statement. The lowest percentage pertained to the understanding of the words and expressions in the written information (question 4), and the highest percentage pertained to how the treatment would be carried out and followed up (question 5).

The physicians’ ratings on a group level were lower than their patients’ for all the questions, and the percentage of ‘fully agree’ ranged between 42.2 and 71.7%. As with the patients, the lowest percentage pertained to the patient’s understanding of the words and expressions in the written information (question 4). However, the highest percentage who fully agreed pertained to the patient’s understanding of the purpose of the clinical trial (question 7). Since only six physicians informed three or more patients, no intra-physician scoring could be performed.

Table 5A shows the paired analyses of the percentage of patients and physicians who fully agree with the seven statements regarding the patients’ perceived understanding (7Q-PAT and 7Q-PHYS). One patient had not answered one of the seven questions and one physician had not answered two other questions, which led to a total of one pair for three different questions being excluded from this pair-wise analysis. For each statement the concordance of ‘fully agree’ was compared for the patient–physician pair (7Q-PAT and 7Q-PHYS).

**Table 5:** Percentage of patients and physicians who fully agree with the statement (*A*) and concordance of patients’ and physicians’ estimation of patient understanding of information (*B*)

		A		B				
	Patient’s/physician’s questions	Patient fully agrees (%)*	Physician fully agrees (%)*	Number of pairs*	Number of concordant pairs	Only patient fully agrees	Only physician fully agrees	McNemar’s test *P* value
1.	I/the patient understood the oral information completely	82.6	56.5	46	26	16	4	0.012
2.	I/the patient understood the written information completely	80.0	51.1	45	26	16	3	0.004
3.	I/the patient understood all words and expressions that the physician used during the oral information	78.3	52.2	46	24	17	5	0.017
4.	I/the patient understood all words and expressions in the written information	64.4	42.2	45	21	17	7	0.064
5.	I/the patient understood how the treatment would be carried out and followed up	91.3	65.2	46	28	15	3	0.008
6.	I/the patient understood what side effects the treatment may have	88.9	60.0	45	26	16	3	0.004
7.	I/ the patient understood the purpose of the research study	87.0	71.7	46	27	13	6	0.167

*If either patient or physician had not answered the question the pair was discarded from both *A* and *B*

Between 46.7 and 60.9% of the pairs for each question were in concordance with respect to whether they fully agreed with the statement or not. For the discordant pairs, there was a higher number where only the patient had rated ‘fully agree’, compared to the number where only the physician had rated ‘fully agree’, for all seven questions. This difference was significant for five of the questions (questions number 1, 2, 3, 5 and 6; Table 5B).

Overall, there was no correlation between the number of ‘fully agree’ for the seven questions of patients’ rating of perceived understanding (7Q-PAT and 7Q-PHYS) and their Q-PUR score, whether measured with 12, six or three questions (all *P* values ≥ 0.47). The percentage of correct answers for each question ranged between 65.2 and 97.8% on a group level (Table 3). Neither was there any correlation between the physicians’ rating of a patient’s understanding and that patient’s Q-PUR knowledge score, whether measured with 12, six or three questions (all *P* values ≥ 0.25).

The concordance between whether or not the patients answered ‘fully agree’ on question 7 (‘I/the patient understood the purpose of the research study’) and scored three out of the three selected Q-PUR questions on study purpose (*n* = 27) was somewhat higher than for the physicians (*n* = 22). However, for discordant pairs patients were significantly more likely to overestimate rather than underestimate their understanding of the trial in relation to their factual knowledge, as measured by the three selected Q-PUR questions (*P* = 0.019). There was no significant direction for the discordant results between whether or not the physicians answered ‘fully agree’ to question 7 and their patient scored three out of three selected Q-PUR questions (*P* = 0.54).

To explore whether or not demographic data may have influenced the results, these factors were compared with the results of both the assessments (7Q-PAT and 7Q-PHYS) and the Q-PUR score. There was no significant association between the patients’ educational level and their rating of their understanding in 7Q-PAT (*P*_trend_ = 0.64), or between the patients’ educational level and the physicians’ rating of their understanding in 7Q-PHYS (*P*_trend_ = 0.75). There were also no significant differences in Q-PUR score due to gender, age or whether the patient or a next of kin had previously participated in a clinical trial. No attempt at sub-group analysis was made due to small numbers. The only factor that made a significant difference was educational level. Patients with higher education scored significantly higher on Q-PUR (Table 6).

**Table 6:** Median scores for Q-PUR questions for all patients and in relation to education level

Educational level	Number of correct answers out of 12 questions, median (range)*	Number of correct answers out of six selected questions, median (range)*	Number of correct answers out of three selected questions, median (range)*
All	11 (4–12)	6 (1–6)	3 (1–3)
Compulsory school	9 (4–11)	5 (1–6)	2 (1–3)
Senior high school	10.5 (6–12)	6 (3–6)	3 (1–3)
College or university	11 (9–12)	6 (4–6)	3 (2–3)
*P* value**	0.001	0.004	0.012

* See Table 3 for the list of the 12, 6, and 3 Q-PUR questions

** Jonckheere-Terpstra Test for Trend

## Discussion

Our study demonstrates that physicians rated patients’ understanding of clinical cancer trial information lower than the patients, and this pattern applied to all questions posed (7Q-PAT and 7Q-PHYS). Neither the patients’ nor their physicians’ ratings correlated with the patients’ factual knowledge, as measured by Q-PUR. The physicians and the patients agreed in their assessment of patient understanding in about half of the patient–physician pairs for every question. When the pairs disagreed, the patients in general rated their understanding as higher than the physicians rated it. Thus, physicians believed that patients understood less than the patients themselves believed.

Physician assessment of patient understanding of clinical trial information, in an individual and pairwise comparison with patients’ own assessment of their understanding, has to our knowledge only been investigated once previously [14]. In that study, however, the physicians were asked to assess patient understanding globally with one question, whereas the patients completed 14 questions. In the present study, both patients and physicians each completed seven questions that mirrored each other. This is a new approach that allows a more detailed examination of problematic areas of patient understanding of clinical trial information.

The present study showed that physicians assess patients’ knowledge lower than the patients’ do. One reason may be that the physicians were more cautious than the patients in scoring full understanding of the information since this is a high standard to meet. Physicians may also be conscious of a number of details that they have not informed the patient about because of lack of time or information overload for the patient or because the physician deemed these details to be less important. Patients, on the other hand, may be unaware of what facts they do not know, and therefore consider themselves to understand completely.

Furthermore, there was no definition of what ‘fully’ agree implied compared to ‘partly’ or ‘not fully’ agree, which might have impacted on the results [20]. This constitutes a limitation. The groups ‘partly’ and ‘not fully’ were merged for statistical reasons, which made this problem smaller. However, the lack of a definition for ‘fully’ understand might still be a problem. On the other hand, the vast majority of patients considered that they fully understand the information regarding all the subjective questions (7Q-PAT), and the scores for factual knowledge as measured by Q-PUR were high for almost all of them. None of the patients or physicians had indicated ‘disagree’, which was the lowest alternative for level of understanding. Altogether, this may be considered to reflect an adequate level of understanding and knowledge. Moreover, there exists no consensus in the literature of what is ‘good enough’ patient understanding for being able to make an informed decision on participation in a clinical cancer trial [21]. The aim is often described as true or completely informed consent, but this may not be a realistic goal.

In order to evaluate whether the ratings of the patients’ understanding were correct, an objective measurement of the patients’ factual knowledge was warranted. There were a limited number of validated, non-trial-specific questionnaires available for cancer at the time the pilot study was planned. The two most relevant questionnaires were the ‘Quality of Informed Consent’ (QuIC) [22] and the ‘Questionnaire—Patient Understanding of Research’ (Q-PUR) [18]. The QuIC includes sections for patients on both perceived understanding (14 questions) and factual knowledge (20 questions). However, the QuIC has no mirroring questions for physicians, which was an important part of the present study. The 14 QuIC patient questions on perceived understanding were deemed too extensive to be converted into mirroring questions for physicians. To keep the number of questions down, Q-PUR was chosen. In the present study, the feasibility and acceptability of the questionnaires were high, and 100% of the questionnaires were returned by patients and physicians. This high return rate was probably due to the small number of patients included in the pilot study, as well as the small scale of the two research sites. The fact that patients had their own personal research nurse who could remind them if they forgot to return the questionnaires probably also contributed to the high return rate.

However, this study gives no support to a correlation between the questions regarding perceived patient understanding (7Q-PAT and 7Q-PHYS) and the factual knowledge questionnaire (Q-PUR). A reason for this finding could be that the content of the questions regarding perceived understanding (7Q-PAT and 7Q-PHYS) does not fully match the Q-PUR questions. Questions 1–4 can be argued to be general enough to match the Q-PUR as a whole (Table 2). Questions 5 and 6 in particular do not have any directly corresponding Q-PUR questions. Only question 7 (‘I/the patient understood the purpose of the research study’) has three specific equivalent Q-PUR questions (questions 1, 4 and 6). Q-PUR as a whole does not give a direct answer to the subjective questions in this study (7Q-PAT and 7Q-PHYS) and may not be sufficiently correlated to be a useful measure of factual knowledge in this context.

The only demographic factor associated with the Q-PUR score was education. A higher educational level was significantly associated with a higher Q-PUR score, which is in line with Hutchison’s results when testing the instrument [18]. Other studies using different instruments have also shown an association between educational level and knowledge [12, 14, 23, 24]. On the other hand, in two studies using the QuIC, a higher education was not associated with better scores [25, 26].

The recruitment window spanned over 4 years. Potential reasons for this long time span are that the study was conducted at two relatively small sites, where each physician was only allowed to participate with five patients, which impaired recruitment speed. Further, changes of research nurses may have contributed to the slow recruitment. Since this was an exploratory pilot study, the number of participants was small, which makes it harder to achieve statistical significance, and no formal a priori power calculation was carried out. Nominal *P* values were presented without adjustment for multiple testing, which may have led to false positive findings, and the findings must therefore be validated in an independent setting [19].

Considering the generalisability of the findings of this study, cancer patients are recruited to clinical trials both in the palliative and in curative/adjuvant settings. This pilot study therefore included clinical trials with a wide range of different diagnoses and settings, in order to capture all different aspects. Since there was no information on the specific trial each patient had been offered, it was not possible to elucidate whether patient understanding differed between the palliative and the curative/adjuvant settings. Further, no information on whether they accepted or declined participation was available. These issues may be addressed if a larger study is to be conducted. However, the aim of the study was to investigate patient understanding of clinical trial information, irrespective of type of trial and participation outcome.

Further, 70% of the physicians were male, while 80% of the patients were female. The high proportion of female patients reflects the fact that breast and ovarian cancer trials were most actively recruiting patients at the time of this study, although several different cancer types were covered. The education level of the patients was high, and 50% of them had a college education, which may reflect the fact that Lund is a University town. For Sweden as a whole, 27% of the population has a university education [27]. Malmö is the third largest city in Sweden with a wide range of socio-economic backgrounds. There are many highly educated people as well as many immigrants with various degrees of education, but as the patients had to be able to speak and read Swedish to be included in this study there were no non-Swedish-speaking patients. These facts suggest there may be a bias towards inclusion of better-educated Swedish-speaking female patients. Further, we have no data on patients who were never invited to participate in the study. These patients may differ from those who the research nurse decided to approach, whereof all agreed to participation. Our sample may therefore not be generalisable to the underlying population of cancer patients.

Although the new questionnaires with mirroring questions were not validated (7Q-PAT and 7Q-PHYS), the content validity was addressed in several ways. Firstly, we chose the questions based on the results from our previous research exploring patients’ preferences [15, 16]. Thereafter, research nurses and physicians tested the questionnaire, and the questions were adjusted according to their feedback. Regarding the Q-PUR, the English version of Q-PUR is validated and tested for reliability [18], although the Swedish version of the questionnaire is not. Furthermore, the translation process may have benefitted from also using, for example, the EORTC quality of life group translation procedure [28].

Taken together, this exploratory pilot study had limitations in different areas, such as limited study size, gender and educational imbalance between patients and physicians on a group level, and a non-validated questionnaire. However, the study has several strengths. The time between information disclosure and measurement of understanding was very short. Usually it took place immediately after the information disclosure for both patients and physicians. This ensured that the risk of memory errors was minimised, which is particularly important for the physicians who include many patients in trials. The greatest strength may be that the patient and physician questionnaires (7Q-PAT and 7Q-PHYS) had mirroring questions and were collected pairwise, which enabled paired analyses. To our knowledge, only one previous study has investigated physicians’ assessment of patient understanding of research [14]. This research field thus needs to be further elucidated.

Our results indicate a gap between patients’ perceived understanding and physicians’ assessment of patient understanding. Studies have also shown that patients may base their decision to participate in a clinical trial on inadequate facts and misunderstandings [4, 29, 30], although they consider themselves to understand the trial information. In order to improve patient understanding of clinical trials, several kinds of interventions have been shown to be effective: enhanced consent documents, decisional aids and extended consent conversations seem to be of particular interest [9, 31]. To optimise the informed consent process, the physicians need to assess better what each patient understands in order to tailor their information better and individually. To achieve this tailoring and to minimise the gap between patients’ perceived understanding and physicians’ assessment of their patients’ understanding, physicians need to improve their communication skills [32, 33].

## Conclusions

This exploratory pilot study indicates a gap between patients and physicians, where the physicians rated their patients’ understanding lower than the patients themselves for all questions on subjective understanding. However, neither the patients’ nor their physicians’ ratings were correlated with the patients’ factual knowledge, as measured by Q-PUR. These findings need validation in an independent setting, with an improved instrument with mirroring questions and a better-matched measurement of patients’ factual knowledge. Nevertheless, these results suggest that physicians need to improve their ability to assess patient understanding of clinical trial information, in order to be able to tailor the patient information individually.

## Data Availability

The datasets generated and/or analysed during the current study are not publicly available.

## Abbreviations

Q-PUR: Questionnaire—Patient Understanding of Research
QuIC: Quality of Informed Consent
SPSS: Statistical Package for the Social Sciences

## References

[1] WMA (2013). World Medical Association Declaration of Helsinki: ethical principles for medical research involving human subjects. JAMA.

[2] Cox K (2002). Informed consent and decision-making: patients' experiences of the process of recruitment to phases I and II anti-cancer drug trials. Patient Educ Couns.

[3] Jenkins V, Solis-Trapala I, Langridge C, Catt S, Talbot DC, Fallowfield LJ (2011). What oncologists believe they said and what patients believe they heard: an analysis of phase I trial discussions. J Clin Oncol.

[4] Dellson P, Nilsson K, Jernstrom H, Carlsson C (2018). Patients' reasoning regarding the decision to participate in clinical cancer trials: an interview study. Trials..

[5] Jefford M, Moore R (2008). Improvement of informed consent and the quality of consent documents. Lancet Oncol.

[6] del Carmen MG, Joffe S (2005). Informed consent for medical treatment and research: a review. Oncologist..

[7] Olson DP, Windish DM (2010). Communication discrepancies between physicians and hospitalized patients. Arch Intern Med.

[8] Flory J, Emanuel E (2004). Interventions to improve research participants' understanding in informed consent for research: a systematic review. JAMA..

[9] Nishimura A, Carey J, Erwin PJ, Tilburt JC, Murad MH, McCormick JB (2013). Improving understanding in the research informed consent process: a systematic review of 54 interventions tested in randomized control trials. BMC Med Ethics.

[10] Kao C.Y., Aranda S., Krishnasamy M., Hamilton B. (2016). Interventions to improve patient understanding of cancer clinical trial participation: a systematic review. European Journal of Cancer Care.

[11] Nathe JM, Krakow EF (2019). The challenges of informed consent in high-stakes, randomized oncology trials: a systematic review. MDM Policy Pract.

[12] Biedrzycki BA (2011). Research information knowledge, perceived adequacy, and understanding in cancer clinical trial participants. Oncol Nurs Forum.

[13] Ellis PM, Butow PN, Tattersall MH, Dunn SM, Houssami N (2001). Randomized clinical trials in oncology: understanding and attitudes predict willingness to participate. J Clin Oncol.

[14] Joffe S, Cook EF, Cleary PD, Clark JW, Weeks JC (2001). Quality of informed consent in cancer clinical trials: a cross-sectional survey. Lancet..

[15] Dellson P, Nilbert M, Bendahl PO, Malmstrom P, Carlsson C (2011). Towards optimised information about clinical trials; identification and validation of key issues in collaboration with cancer patient advocates. Eur J Cancer Care (Engl)..

[16] Dellson P, Nilbert M, Carlsson C (2016). Patient representatives' views on patient information in clinical cancer trials. BMC Health Serv Res.

[17] Cox AC, Fallowfield LJ, Jenkins VA (2006). Communication and informed consent in phase 1 trials: a review of the literature. Support Care Cancer.

[18] Hutchison C, Cowan C, Paul J (2007). Patient understanding of research: developing and testing of a new questionnaire. Eur J Cancer Care (Engl)..

[19] Bender R, Lange S (2001). Adjusting for multiple testing--when and how?. J Clin Epidemiol.

[20] Schaeffer N (1991). Hardly ever or constantly. Group comparisons using vague quantifiers. Public Opin Q.

[21] Sand K, Kaasa S, Loge JH (2010). The understanding of informed consent information—definitions and measurements in empirical studies. AJOB Prim Res.

[22] Joffe S, Cook EF, Cleary PD, Clark JW, Weeks JC (2001). Quality of informed consent: a new measure of understanding among research subjects. J Natl Cancer Inst.

[23] Miller CK, O'Donnell DC, Searight HR, Barbarash RA (1996). The Deaconess Informed Consent Comprehension Test: an assessment tool for clinical research subjects. Pharmacotherapy..

[24] Cameron P, Pond GR, Xu RY, Ellis PM, Goffin JR (2013). A comparison of patient knowledge of clinical trials and trialist priorities. Curr Oncol.

[25] Bergenmar M, Johansson H, Wilking N (2011). Levels of knowledge and perceived understanding among participants in cancer clinical trials - factors related to the informed consent procedure. Clin Trials.

[26] Brandberg Y, Johansson H, Bergenmar M (2016). Patients' knowledge and perceived understanding - Associations with consenting to participate in cancer clinical trials. Contemp Clin Trials Commun.

[27] Educational attainment of the population in 2017 [http://www.scb.se/en/finding-statistics/statistics-by-subject-area/education-and-research/education-of-the-population/educational-attainment-of-the-population/pong/statistical-news/educational-attainment-of-the-population-in-2017/]. Accessed 10 Dec 2018.

[28] EORTC quality of life group translation procedure [http://www.eortc.org/app/uploads/sites/2/2018/02/translation_manual_2017.pdf]. Accessed 10 Dec 2018.

[29] Behrendt C, Golz T, Roesler C, Bertz H, Wunsch A (2011). What do our patients understand about their trial participation? Assessing patients' understanding of their informed consent consultation about randomised clinical trials. J Med Ethics.

[30] Bergenmar M, Molin C, Wilking N, Brandberg Y (2008). Knowledge and understanding among cancer patients consenting to participate in clinical trials. Eur J Cancer.

[31] Sundaresan P, Ager B, Turner S, Costa D, Kneebone A, Pearse M, Woo H, Tesson S, Juraskova I, Butow P (2017). A randomised controlled trial evaluating the utility of a patient Decision Aid to improve clinical trial (RAVES 08.03) related decision-making. Radiother Oncol.

[32] Wade J, Donovan JL, Lane JA, Neal DE, Hamdy FC (2009). It's not just what you say, it's also how you say it: opening the 'black box' of informed consent appointments in randomised controlled trials. Soc Sci Med.

[33] Hietanen PS, Aro AR, Holli KA, Schreck M, Peura A, Joensuu HT (2007). A short communication course for physicians improves the quality of patient information in a clinical trial. Acta Oncol.

